# Evaluation of anti-malaria potency of wild and genetically modified *Enterobacter cloacae* expressing effector proteins in *Anopheles stephensi*

**DOI:** 10.1186/s13071-022-05183-0

**Published:** 2022-02-19

**Authors:** Hossein Dehghan, Seyed Hassan Mosa-Kazemi, Bagher Yakhchali, Naseh Maleki-Ravasan, Hassan Vatandoost, Mohammad Ali Oshaghi

**Affiliations:** 1 Department of Public Health, School of Public Health, Jiroft University of Medical Sciences, Jiroft, Iran; 2 Department of Medical Entomology and Vector Control, School of Public Health, Tehran University of Medical Sciences, Tehran, Iran; 3 Department Industrial and of Environmental Biotechnology, National Institute of Genetic Engineering and Biotechnology, Tehran, Iran; 4 Malaria and Vector Research Group, Biotechnology Research Center, Pasteur Institute of Iran, Tehran, Iran; 5 Department of Chemical Pollutants and Pesticides, Institute for Environmental Research, Tehran University of Medical Sciences, Tehran, Iran

**Keywords:** *Anopheles stephensi*, Malaria, *Enterobacter cloacae*, Paratransgenesis, Defensin, Scorpine

## Abstract

**Background:**

Malaria is one of the most lethal infectious diseases in tropical and subtropical areas of the world. Paratransgenesis using symbiotic bacteria offers a sustainable and environmentally friendly strategy to combat this disease. In the study reported here, we evaluated the disruption of malaria transmission in the *Anopheles stephensi-Plasmodium berghei* assemblage using the wild-type (WT) and three modified strains of the insect gut bacterium, *Enterobacter cloacae*.

**Methods:**

The assay was carried out using the *E. cloacae dissolvens* WT and three engineered strains (expressing green fluorescent protein-defensin (GFP-D), scorpine-HasA (S-HasA) and HasA only, respectively). Cotton wool soaked in a solution of 5% (wt/vol) fructose + red dye (1/50 ml) laced with one of the bacterial strains (1 × 10^9^cells/ml) was placed overnight in cages containing female *An. stephensi* mosquitoes (age: 3–5 days). Each group of sugar-fed mosquitoes was then starved for 4–6 h, following which time they were allowed to blood-feed on *P. berghei*–infected mice for 20 min in the dark at 17–20 °C. The blood-fed mosquitoes were kept at 19 ± 1 °C and 80 ± 5% relative humidity, and parasite infection was measured by midgut dissection and oocyst counting 10 days post-infection (dpi).

**Results:**

Exposure to both WT and genetically modified *E. cloacae dissolvens* strains significantly (*P* < 0.0001) disrupted *P. berghei* development in the midgut of *An. stephensi*, in comparison with the control group. The mean parasite inhibition of *E. cloacae*^WT^, *E. cloacae*^HasA^, *E. cloacae*^S−HasA^ and *E. cloacae*^GFP−D^ was measured as 72, 86, 92.5 and 92.8 respectively.

**Conclusions:**

The WT and modified strains of *E. cloacae* have the potential to abolish oocyst development by providing a physical barrier or through the excretion of intrinsic effector molecules. These findings reinforce the case for the use of either WT or genetically modified strains of *E. cloacae* bacteria as a powerful tool to combat malaria.

**Graphical Abstract:**

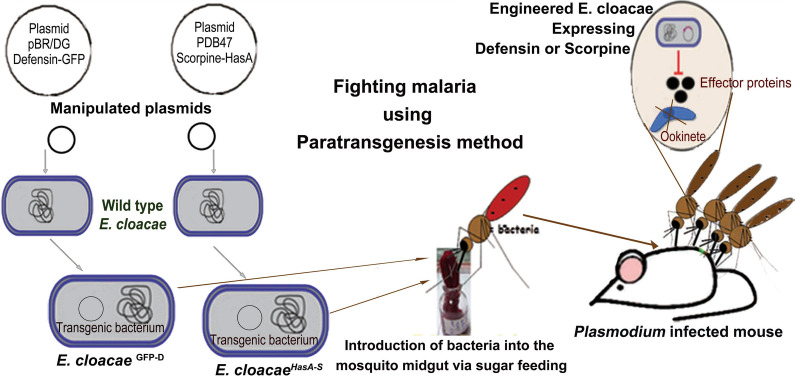

**Supplementary Information:**

The online version contains supplementary material available at 10.1186/s13071-022-05183-0.

## Background

Of the world's vector-borne diseases, malaria causes the greatest health concern, with a reported 229 million cases and 409,000 deaths globally in 2019 [1]. The *Plasmodium* parasite is the causative agent of malaria, and the female *Anopheles* mosquito is the vector of the disease. *Anopheles stephensi* is the main malaria vector throughout its range from Asia to the Horn of Africa [2–5]. Therefore, programs aimed at controlling *A. stephensi* populations and limiting the ability of these mosquitoes to transmit *Plasmodium* (refractory mosquitoes) have the potential to reduce the burden of malaria disease [6].

Currently, the most common methods of mosquito control are indoor residual spraying (IRS) and insecticide-treated nets (ITNs) [1, 7]. However, novel and innovative control measures are urgently needed due to emerging insecticide resistance in malaria vectors, particularly in *A. stephensi* [8–10], and increasing eco-environmental concerns about the off-target effects of insecticide use [11–15]. Transmission-blocking strategies (TBS) have recently been proposed as a potential means of malaria control, with an increased emphasis on inhibiting the development of the *Plasmodium* parasite in the vector mosquito [16–19]. Gametocytocidal drugs, transmission-blocking vaccines and the replacement of wild mosquitoes with refractory mosquitoes are currently the most important methods used in TBS. The last method consists of genetically manipulating *Anopheles* mosquitoes to render them refractory to *Plasmodium* parasite development. This is accomplished using anti-plasmodium molecules (transgenesis) [20], naturally refractory mosquitoes [21], artificial gene-drive mechanisms [22, 23] and/or micro-symbionts genetically modified by effector molecules that are reintroduced into the wild mosquito population (paratransgenesis) [6, 12, 24, 25].

During its development in its invertebrate hosts (vectors), the *Plasmodium* parasite undergoes a decreasing population trend, from 10^3^–10^4^ gametocytes to 10^2^–10^3^ motile ookinetes and, finally, to ≤ 5 oocysts [26, 27]. This bottleneck could be considered a prime target for intervention and the blocking of parasite transmission [28, 29]. The main factors creating the bottleneck include gut digestive enzymes, the mosquito's immune responses and intestinal microbial flora. The intestinal microbial flora plays a vital role in blocking parasite development in the *Anopheles* midgut. This effect is exerted directly by the proliferation of bacteria after a blood meal, simultaneously with the development of the ookinete stage, and indirectly via the expression of antimicrobial genes [30–36].

To date, a number of different symbiotic bacteria have been suggested for use in a paratransgenesis strategy for combating malaria. *Serratia* AS1 (isolated from *Anopheles* spp.), *Asaia* sp. (isolated from *Anopheles gambiae*, *An. stephensi*, *Aedes albopictus* and *Aedes aegypti)* and *Pantoea agglomerans* (isolated from *An. stephensi, An. gambiae* and *Anopheles funestus*) are the bacterial species most commonly used for the paratransgenic control of malaria [11, 25, 27, 31, 37–41]. These genetically modified bacteria could potentially eliminate the development of the *Plasmodium* parasite in the *Anopheles* midgut by expressing anti-*Plasmodium* molecules. However, due to convergent evolution, the wild-type (WT) bacteria have shown limited intrinsic antiparasitic activities in the mosquito midgut [27, 42].

*Enterobacter cloacae,* a Gram-negative, facultative anaerobic, rod-shaped bacterium, has been found to be a component of the microflora of *An. stephensi* [43, 44], *Anopheles albimanus* [45], *Ae. albopictus* and *Ae. aegypti* [46], as well as of other medically important insects [47, 48]. This bacterium has been shown to have an innate blocking effect on *Plasmodium* development and could potentially limit *P. berghei* and *P. falciparum* development in *An. stephensi* by markedly increasing the population, thereby leading to stimulation of the mosquito immune system and expression of immune response compounds, such as serine protease inhibitors (SRPN6) [49]. These innate features suggest that *E. cloacae* is a suitable candidate for paratransgenesis studies against the malaria parasite.

Scorpine is a small cationic antimicrobial peptide (AMP) found in the venom of the scorpion *Pandinus imperator* with anti-plasmodial and anti-bacterial activity, and also a strong inhibition of dengue 2 virus (DENV-2) infection [50–52]. Defensins are small cysteine-rich cationic proteins that are found in plants, vertebrates and invertebrates [53–55]. Scorpine has been used as effector molecule against malaria parasites in paratransgenic mosquitoes carrying scorpine-secreting bacteria or fungi [11, 27, 56]. The aim of the present study was to evaluate the transmission-blocking potential of WT and engineered strains of *E. cloacae* expressing defensin and scorpine effector molecules to block *P. berghei* development in *An. stephensi*.

## Methods

### Mosquito rearing

*Anopheles stephensi*, Beech strain, was used in this study. The strain was originally collected in Pakistan as an additional type of the SDA500 strain and was kindly provided in 2005 by Professor P.F. Billingsley, Sanaria, Inc. [57]. Mosquitoes were maintained on a 5% (wt/vol) fructose solution at 27 ± 1 °C and 65 ± 5% relative humidity (RH) under a 12:12 dark:light (D:L) photoperiod. All mosquito-rearing facilities were provided by Tehran University of Medical Sciences, School of Public Health.

### Maintenance of parasite life-cycle

**Fig. 1:**
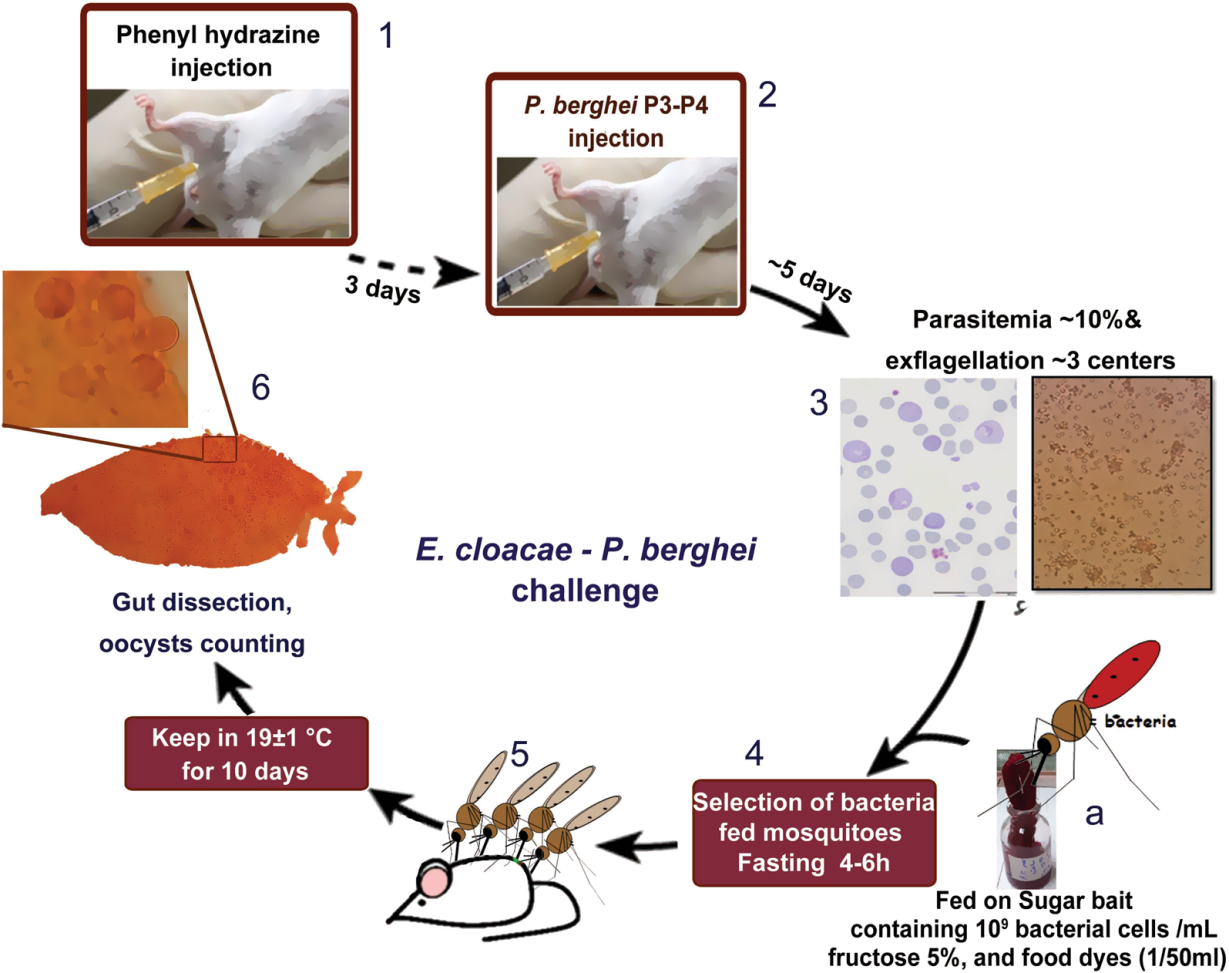
Schematic illustration of the transmission blocking assay. Steps **1–3**: Infection of mouse with *Plasmodium berghei* ANKA strain clone 2.34 (**a** shows the introduction of bacteria into the mosquito midgut via sugar bait feeding). Steps **4**,** 5** Sugar-fed mosquitoes are fasted for 4–6 h (**4**), following which they blood-feed on a* P. berghei*-infected mouse (**5**). Step **6** Dissection of the mosquito midgut, staining with 0.5% mercurochrome and counting of the infective oocysts under the light microscope

BALB/c mice aged 6 weeks (weight 8–20 g) were used in this study. The mouse colonies were maintained in an animal house at 40–50% RH and 24 ± 1 °C. The *P. berghei* ANKA strain was used, specifically clone 2.34 (a gift from Prof. Marcelo Jacob-Lorena, Johns Hopkins Bloomberg School of Public Health, Baltimore, MD, USA). Parasites were maintained using previously described protocols [58, 59]. Briefly, the parasites were maintained in female BALB/c mice by serial mechanical passages (3 or 4 passages). To maintain gametocyte infectivity to mosquitoes, in direct passages, hyper-reticulocytosis was induced 3 days before infection by treating each mouse with 100 μl 1% phenylhydrazinium chloride (PH) (Sigma-Aldrich, St. Louis, MO, USA) administered intraperitoneally (ip; 10 mg/ml in phosphate-buffered saline [PBS]) per 10 g mouse body weight. Parasitemia was monitored in Giemsa-stained tail-blood smears. Exflagellation was examined at 5–6 days post-infection (dpi) (dose of injected parasite: approx. 10^4^) by mixing a drop of infected blood (4-5 µl) with 20–25 μl ookinete culture medium (RPMI 1640 medium containing L-glutamine and 25 mM HEPES, 2 g/l NaHCO, 50 mg/l hypoxanthine, 50,000 U/l penicillin and 50 mg/l streptomycin; pH 8.3, filter sterilized). Mice with approximately three exflagellation centers in each field of 40× microscopic magnification were used in the transmission blocking assay (Fig. 1).

### Transformation of bacteria

**Fig. 2:**
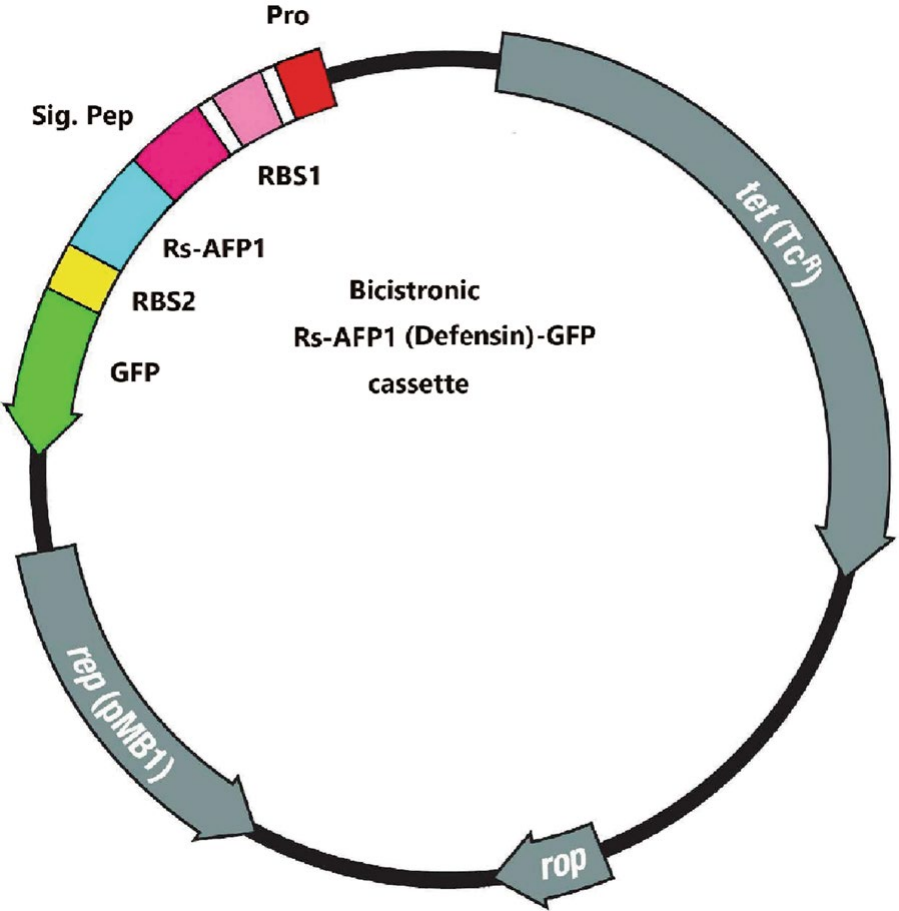
Schematic illustration of the defensin-GFP (DG) construct and its position in the pBR322 plasmid.* Abbreviations*: Pro, promotor; RBS1 and RBS2, ribosomal binding site numbers 1 and 2, respectively; Sig Pep, signal peptide; Rs-AP1, defensin; GFP, green fluorescent protein. The EcoRI and Pst1 restriction sites are at the 5′ and 3′ end of the construct

**Fig. 3:**
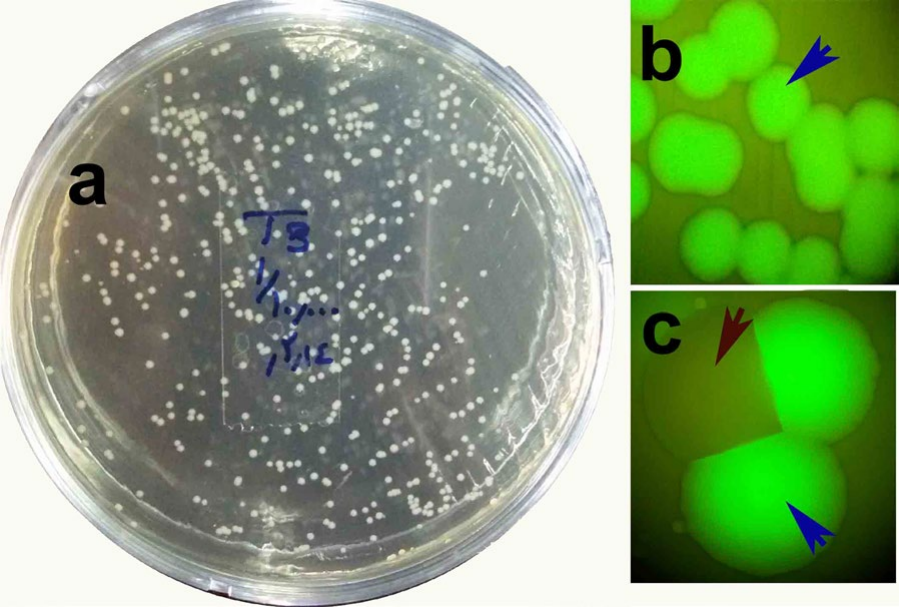
Wild-type (**a**) and recombinant *E. cloacae dissolvens* expressing GFP (**b**, **c**)

The *E. cloacae dissolvens* subspecies used in this study was originally isolated by sampling the midgut microflora of the sand fly *Phlebotomus papatasi*, found in the main zoonotic cutaneous leishmaniasis foci in central Iran. The procedures used for isolating, characterizing and identifying this bacterium have been outlined previously [47]. We genetically engineered two different *E. cloacae* strains to produce: (1) a strain containing defensin (a peptide isolated from radish seeds, Rs-AFP [60]) plus green fluorescent protein (GFP), referred to here as *E. cloacae*^GFP−D^), and (2) a strain containing Scorpine-HasA (scorpion *Pandinus imperator* venom) plus HasA, a heme-binding protein as an exporting system [61], referred to here as *E. cloacae*^S−HasA^. Defensin and scorpine proteins are anti-malarial effector molecules with different killing mechanisms. As a control, we also genetically engineered a strain of *E. cloacae* to produce only HasA, referred to here as *E. cloacae*^HasA^. The transgenic *E. cloacae*^GFP−D^ strain with the originally manipulated pBR322 plasmid was used in this study. The engineered pBR322 plasmid containing the defensin gene as effector molecule plus a GFP marker and tetracycline resistance genes, called the pBR/DG plasmid, is maintained in Tehran University of Medical Sciences (Fig. 2). The β-lactamase gene of the plasmid was replaced with the DG construct. A detailed description of the construct is provided in Additional file 1: Dataset S1. The recombinant plasmid was electro-transformed first to the recipient strain of *Escherichia coli*-DH5α and then to *E. cloacae dissolvens* cells. This strain is distinguishable from other similar bacteria colonies its green fluorescence under microscopy (Fig. 3). For construct verification, it was amplified from the pBR/DG plasmid using the forward primer DGFP1 (5′-GGA ATT CAA ATA CAT TCA AAT ATG TAT CCG-3′) and the reverse primer DGFP7 (5′-TTC TGC AGT TAT TAT TTG TAT AGT TCA TCC ATG-3′). The pBR/DG and pBR322 plasmids also were digested at the EcoRI and Pst1 restriction sites which were incorporated at the 5′ and 3′ ends of the construct, respectively. The digested plasmids were electrophoresed in 1% agarose gel (Additional file 2: Fig. S1).

The proteins were subjected to sodium dodecyl sulfate–polyacrylamide gel electrophoresis (SDS-PAGE) to detect the secreted defensin molecules from the *E. cloacae dissolvens* strain carrying the pBR/DG plasmid [62]. For this purpose, the recombinant strain was grown overnight at 37 °C in LB liquid medium containing the tetracycline (12.5 µg/ml) antibiotics. Bacterial cultures were centrifuged at 4000 *g* for 15 min at 4 °C, and the supernatant was collected and separated by 13% gradient SDS-PAGE. The supernatant from WT *E. cloacae dissolvens* was used as a control.

We used two plasmids, including PDB47-Scorpine-HasA and PDB47-HasA hosted in *Serratia* AS1 (a gift from Prof. Marcelo Jacob-Lorena, Johns Hopkins Bloomberg School of Public Health, Baltimore, MD, USA) [11]. The plasmids were extracted and transferred into the WT *E. cloacae dissolvens*.

### Transmission-blocking assay

The transmission-blocking assay was carried out using WT/engineered bacteria, including the ampicillin-resistant *E. cloacae* WT, the ampicillin- and tetracycline-resistant *E. cloacae*^GFP−D^ strain and the ampicillin- and apramycin-resistant *E*. *cloacae*^HasA^ and *E. cloacae*^S−HasA^ strains. The bacterial strains were cultured in BHI broth at 37 °C, and antibiotics, including ampicillin (100 μg/ml), tetracycline (12.5 μg/ml) and apramycin (80 μg/mL) were added to the media based on their antibiotic resistance patterns. After overnight growth, the bacteria were harvested by centrifugation (3000 *g*, 10 min), washed twice in sterile PBS and resuspended (5% wt/vol)in a sterile fructose and red dye (1/50 ml) solution (Nilgoon®, Tehran, Iran) to obtain a concentration of 10^9^ cells/ml.

Female mosquitoes aged 3–5 days were placed in five groups, each containing 25 females, and fed on sterile cotton wool soaked in the fructose + dye solution, with or without bacterial cells, for 24 h. As each mosquito became satiated with the sugar solution, as identified by the red dye, they were separated via a sucking tube and transferred into another cage where they were starved for 4–6 h prior to being allowed to feed on infected blood. Each of the five groups of mosquitoes (each comprising 15–20 females) were fed on the same *P. berghei*-infected mouse. The infected blood-feeding process was carried out at 17–20 °C for 20 min, and the infected blood-fed mosquitoes were maintained on 5% fructose/0.05% para-aminobenzoic acid (PABA) at 19 ± 1 °C and 75 ± 5% RH under a 12:12 D:L photoperiod. Data were pooled from four biological replicates.

In total, we dissected midguts from 248 mosquitoes (45–51 females in each group) at 10 days post-infected blood-feed (dpi); these midguts were stained with 0.5% (wt/vol) mercurochrome (Aldrich–Sigma). *Plasmodium* oocyst development was examined by light microscopy and the oocysts were counted (Fig. 1).

**Fig. 4:**
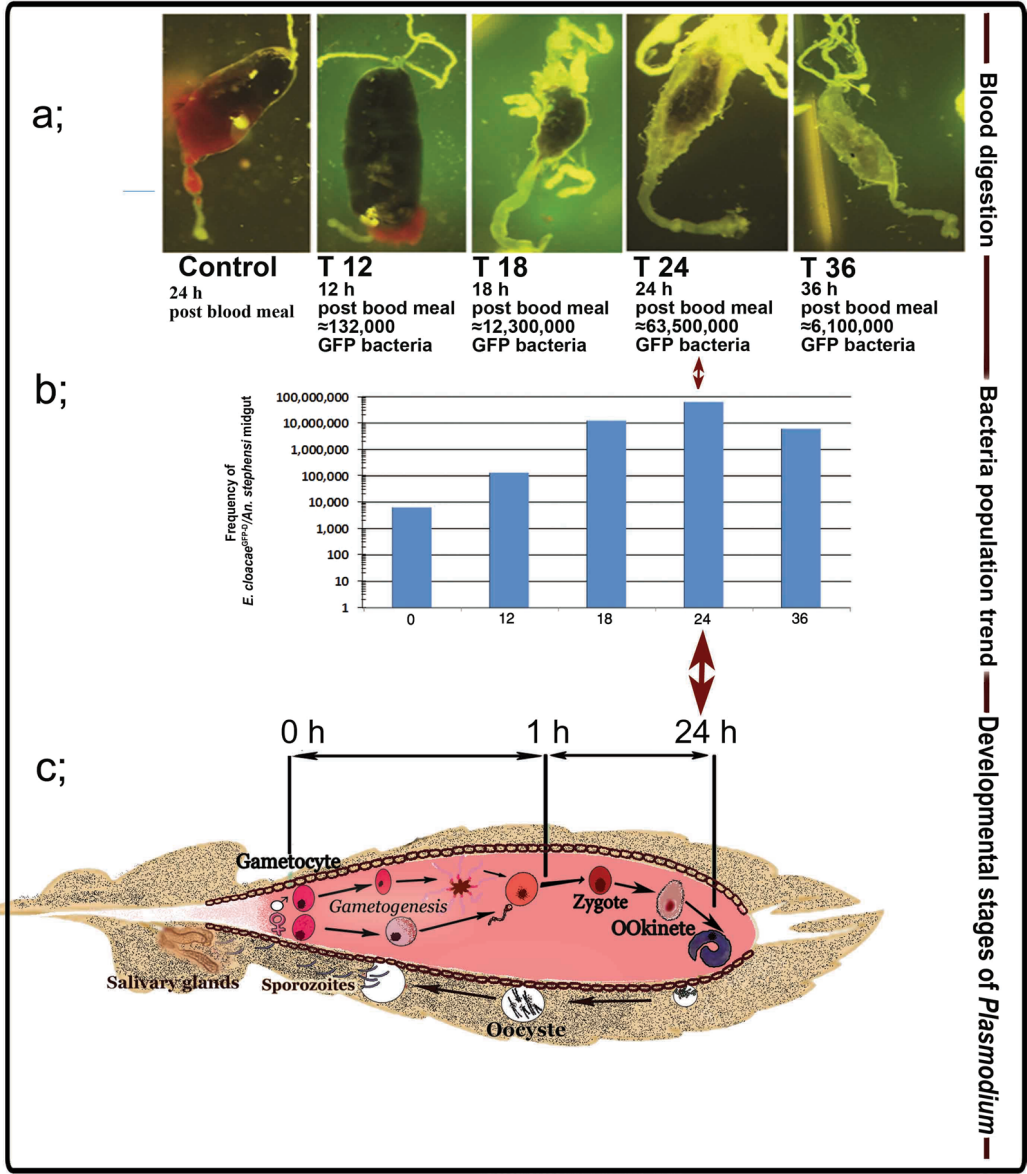
Trends of *E. cloacae* proliferation in the mosquito midgut. **a** Digestion of blood meal in paratransgenic *Anopheles stephensi* over time. **b** The frequency of *E. cloacae*^GFP-D^ in the *An. stephensi* midgut at different times (T) after ingestion of a blood meal. **c** Schematic development of the *Plasmodium* parasite in *Anopheles* midgut shows simultaneous bacterial proliferation and ookinete formation at 18–24 h after a blood meal

A subset of the sugar-fed females infected with bacteria, as well as the blood-fed specimens, were tested for the presence and proliferation of the bacteria by midgut dissection at 0, 12, 18, 24, and 36 h post-blood meal (Fig. 4). The population dynamics of the engineered bacteria colony-forming units (CFUs) were defined by plating serially diluted homogenates of midguts on LB agar plates containing 100 μg/ml of the appropriate antibiotics.

All experiments on the rodents were performed in accordance with the guidelines of the Ethical Board of Tehran University of Medical Sciences (TUMS), Iran.

### Statistical analyses

The normality of data was checked using the Shapiro-Wilks test. Significant differences in oocyst intensities between two samples were analyzed using the Mann–Whitney test. Multiple-sample comparisons were analyzed using the nonparametric Kruskal–Wallis test, and means and medians were compared using Dunn’s test. All statistics were performed using GraphPad Prism version 5.00 for Windows (GraphPad Software, San Diego, CA, USA). A* P*-value  < 0.05 was considered to be statistically significant.

## Results

### Proliferation of bacteria in mosquito midgut

**Fig. 5:**
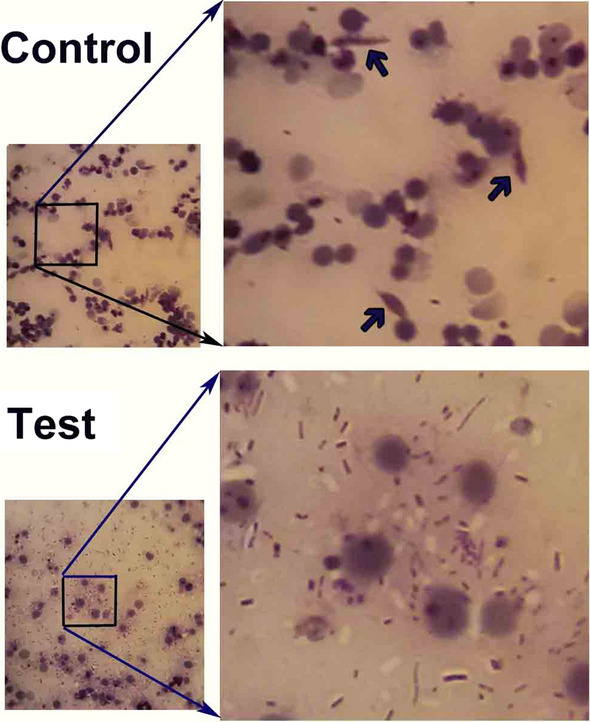
The presence or absence of motile ookinetes (barrel*-*shaped, shown by arrows) of *P. berghei* in the midgut of *An. stephensi* mosquitoes in the control and *E. cloacae*^GFP-D^ (Test) group at 20–24 h after ingestion of an infected blood meal. Upper panels: Presence of *P. berghei* ookinetes are seen in the remains of digested red blood cells in the mosquito midgut in the absence of *E. cloacae* bacteria. Lower panels: Strong proliferation of *E. cloacae*^GFP-D^ bacteria correlates with a lack of ookinetes 20 h after ingestion of an infected blood meal with 10^4^–10^5^ parasites/µl in the midgut. The thick smear was stained by Giemsa. The bacteria in the dissected *An. stephensi* midgut (squares) were transferred to BHI agar plates and found to express GFP under the fluorescent (Fig. 3b) microscope

The dynamics of the engineered bacteria showed that, in general, the bacteria were easily established in the mosquito midgut through feeding the mosquitoes on sugar meals laced with bacteria. The bacteria proliferated strongly in the mosquito midgut after ingestion of a blood meal and became the dominant microflora of the midgut, based on the number of CFUs in the plates. *Enterobacter cloacae* dynamics were monitored at different times post-blood meal by dissection of the midgut and plating of the midgut homogenates on selective antibiotic-containing plates. The number of engineered bacteria increased dramatically, by more than approximately 10,000-fold at 24 h after ingestion of a blood meal (Fig. 4a, b), and the rapid propagation of transgenic *E. cloacae* occurred simultaneously with the development of the ookinete stage of the *Plasmodium* parasite in the mosquito gut (Fig. 4c, 5).

### Transmission blocking assay

The oocyst numbers in the mosquito midguts were counted on 10 dpi. The median values of all strains of WT or transgenic bacteria significantly (*P* < 0.0001) impaired the development of *P. berghei* in the *An. stephensi* midgut, in comparison with the control group (Table 1).

**Table 1:** *P*-value and significance level of *Plasmodium berghei* development inhibition in paratransgenic* Anopheles stephensi* harboring different *Enterobacter cloacae* strains

*Enterobacter cloacae* strains	Without bacteria	WT	HasA	Sco.	Def.
Without bacteria		***	***	***	***
WT	< 0.0001		No	**	**
HasA	< 0.0001	0.176		No	*
Sco.	< 0.0001	0.007	0.051		No
Def.	< 0.0001	0.003	0.020	0.323	

*P*-value < 0.05 was considered to be statistically significant. *, **, *** indicate significant difference at* P* < 0.05,* P* < 0.01,* P* < 0.001, respectively

WT, *Enterbacter cloacae* wild type; HasA, *E*.* cloacae* expressing HasA (*E. cloacae*^HasA^); Sco,* E. cloacae* expressing scorpine and HasA (*E. cloacae*^S−HasA^); Def, *E. cloacae* expressing defensin and green fluorescent protein (*E.cloacae*^GFP−D^)

**Fig. 6:**
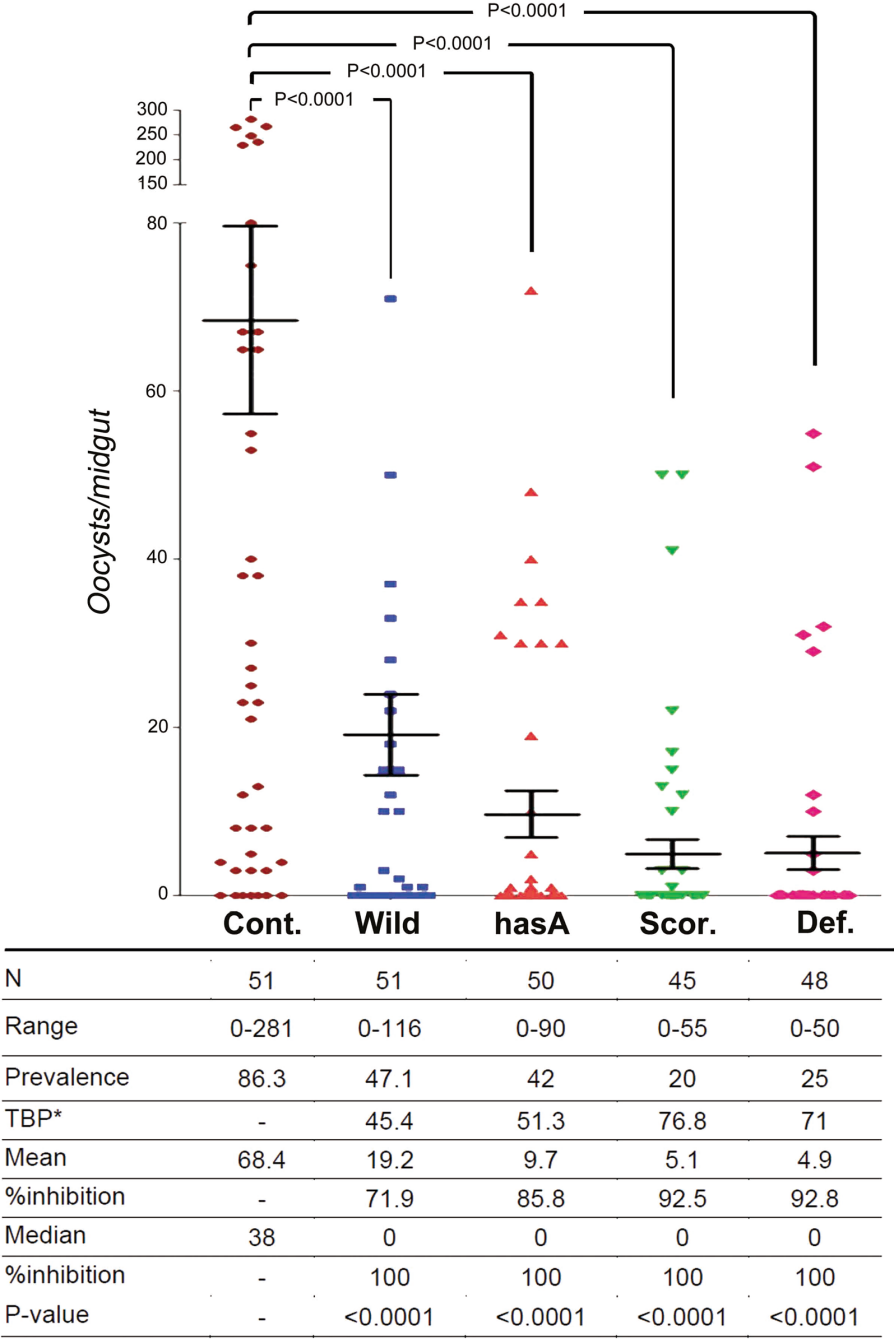
Inhibition of *P. berghei* development in *An. stephensi* by WT and transgenic *E. cloacae* strains. *Anopheles stephensi* mosquitoes were fed on 5% (wt/vol) fructose solution + red food dye supplemented with either phosphate-buffered saline (control) or with WT or transgenic *E. cloacae* strains in five groups. After 8 h, the five groups of mosquitoes were fed on the same *P. berghei*-infected mice. Oocyst numbers were determined 10 days after the infected blood meal. Data were pooled from four biological replicates. Each dot denotes the oocyst number of an individual midgut, and horizontal lines show mean values. %inhibition refers to the inhibition of oocyst formation relative to the control. Mean refers to the mean oocyst number per midgut. Median refers to the median oocyst number per midgut. N is the number of mosquitoes analyzed. Prevalence refers to the percentage of mosquitoes carrying at least one oocyst. Range refers to the range of oocyst numbers per midgut. TBP: 100 − {(prevalence of mosquitoes fed with transgenic *E. cloacae*)/[prevalence of control (− Bacteria) mosquitoes] × 100}. Inhibition refers to the inhibition of oocyst formation relative to the control. *Abbreviations*: Cont, control group without bacteria; Wild, *E. cloacae* WT; HasA, *E*. *cloacae*^HasA^; Scor., *E. cloacae*^S−HasA^; Def., *E. cloacae*^GFP−D^; TBP, transmission blocking potential

The WT strain of *E. cloacae* was found to inhibit oocyst formation by approximately 72%, in comparison with the control group (without bacteria). The transgenic *E. cloacae* strain expressing the HasA protein alone inhibited oocyst formation by 86%, while the transgenic *E. cloacae* strain expressing both the HasA and scorpine proteins inhibited oocyst formation by 92.5%. The transgenic *E. cloacae* strain expressing the defensin protein had the greatest inhibitory effect (92.8%) on oocyst formation. Importantly, the infection prevalence (the percentage of mosquitoes that had ≥ 1 oocysts) was 86.3% in the control group, which was reduced to 47.1, 25 and 20% in paratransgenic mosquitoes with the WT type, GFP-D and S-HasA strains, respectively. The transmission-blocking potential (TBP) index was determined in paratransgenic mosquitoes with the WT, GFP-D and S-HasA strains was determined to be 45.4, 71 and 76.8, respectively (Fig. 6).

## Discussion

The *Plasmodium* parasite is very vulnerable in the mosquito midgut and, consequently, components of the midgut microbiome could negatively affect parasite development in several ways, such as by impairing its development through secreting anti-parasitic compounds, by activating the host immune system and/or by competing with the parasite for available space in the midgut [6, 27]. *Enterbacter cloacae* is a known symbiont of the gut microflora of most *Anopheles* species; as such, it has been suggested as a good candidate for the paratransgenic control of malaria [63]. In our study, transgenic *E. cloacae* showed rapid propagation by 18–24 h after the mosquito consumed the blood meal, suggesting it can block parasite development by competing for the same space as the parasite (Figs. 4c, 5), by a greatly increased expression of anti-plasmodium molecules that lyse the *Plasmodium* parasite in the *Anopheles* midgut (Fig. 6) and by activating the mosquito immune system against the bacteria, which also leads to parasite control [49].

The present study was designed to investigate the efficacy of different strains of *E. cloacae* in disrupting *P. berghei* development, while previous studies have investigated different aspects of *E. cloacae* [45, 49, 63, 64]. In our study, we showed that *E. cloacae* multiplied rapidly in the mosquito midgut, and that by 18–24 h after ingestion of a blood meal it was the dominant species in the midgut microflora. This was shown by the GFP marker and by culturing the mosquito's midgut contents at different times after the blood meal. Similarly, Pumpuni et al. [31] showed that the midgut bacterial load of *An. gambiae* and *An. stephensi* increased by 11–40 fold by 24 h after blood-feeding. Demaio et al. [65] also obtained similar results in *Aedes triseriatus, Culex pipiens* and *Psorophora columbiae*, and Wang et al. [27] reported that the bacterial load of *Plasmodium agglomerans* increased by 200-fold in the *An. stephensi* midgut by 24–48 h after ingestion of a blood meal. The finding of Dehghan et al. [63], that *E. cloacae* was highly stable in a sugar solution, suggested that using sugar bait stations to introduce the transgenic bacteria in the field could be a feasible paratransgenic approach.

It is known that the development of the *Plasmodium* parasite could be affected by the presence of certain bacteria in the microflora of the mosquito midgut. In this study, the interaction of *E. cloacae* and *P. berghei* in vivo led to a significant inhibition of oocyst formation, relative to the control group (*P*-value < 0.0001). This correlates well with the findings of Pumpuni et al. [30] who reported that the presence of 100,000 *Ewingella americana* cells in the mosquito midgut reduced the *P. falciparum* infection rate to zero and those of Gonzalez-Ceron et al. [45] who reported a reduction in the *P. vivax* infection rate in *An. albimanus* in the presence *Serratia marcescens, E. cloacae* and *Enterobacter amnigenus*. In this regard, the coincidence of bacterial multiplication with the ookinete stage in the *Anopheles* gut will affect the bacteria–parasite interaction both directly and indirectly. We showed that transgenic bacteria could overcome the harsh environment and barriers in the *Anopheles* midgut, such as digestive enzymes, to become the dominant component of the gut microflora, leading to an increase in the expression of antiparasitic molecules. This correlates well with the findings of Dong et al. [32], who showed that when the *Chryseobacterium meningosepticum* bacterium entered the *An. gambiae* midgut, it rapidly became a dominant species, indicating the competitive nature of this bacterium in the midgut environment.

The results of this study showed that all of the bacterial strains tested disrupted the development of *P. berghei* to a significant degree, compared with the control group (*P* < 0.0001). Even the *E. cloacae* WT led to significantly impaired parasite development (*P* < 0.0001), indicating the inherent effect of these bacteria in parasite control. The transgenic *E. cloacae* ^GFP−D^ strain, expressing defensin, inhibited parasite development still further compared with the WT (*P* = 0.003), indicating the suppressive effect of defensin, which lyses the parasite inside the mosquito gut [66–68]. The inhibitory effect of scorpine was very similar to that of defensin, and we saw no significant differences in inhibition of oocyst formation between *E. cloacae*^S−HasA^ and *E. cloacae*^GFP−D^ (*P* = 0.051). Similarly, Kokoza et al. [66] expressed cecropin A and defensin A in *Ae. aegypti* mosquitoes to control *P. gallinaceum,* and reported that *Plasmodium* transmission was completely blocked.

Scorpine is an anti-malarial peptide from the venom of the *Pandinus imperator* scorpion and its amino acid sequence is very similar to those of cecropin and defensin, which has led to the suggestion that scorpine might have a similarly inhibitory effect on the *P. berghei* [50]. Indeed, Conde et al. [50] found that it completely inhibited *P. berghei* fertilization and oocyst formation. Wang et al. [11, 27] reported that the symbiotic bacteria, *P. agglomerans* and *Serratia* AS1, transgenically expressing scorpine, could inhibit *P. falciparum* development in *An. gambiae* by 98% and 93%, respectively. The additional expression of HasA protein in the *E. cloacae*^S−HasA^ strain was found to enhance the anti-*Plasmodiun* effectiveness of scorpine. It is possible that HasA could create a membrane pore in the *E. cloacae* wall to allow the direct export of scorpine protein from the bacterial cytoplasm into the mosquito midgut.

Three bacterial species have previously been proposed as candidates for paratransgenetic malaria control: *Serratia* AS1, *Asaia* sp. and *P. agglomerans*; these species transgenically express anti-*Plasmodium* proteins and have been demonstrated to be suitable micro-symbionts in the mosquito midgut [27, 42]. Here, we evaluated a new candidate bacterium, *E. cloacae,* and showed that it has a strong innate control effect on the *Plasmodium* parasite in the mosquito midgut, and that this effect could be enhanced by the transgenic expression of anti-*Plasmodium* proteins.

The symbiotic bacterium *Asaia*, transgenically expressing scorpine, has previously been shown to inhibit *P. berghei* development by 63% in the *An. stephensi* midgut [42]; in comparison, we found that, when expressed in *E. cloacae*, scorpine caused a 92.5% inhibition of oocyst formation. This remarkable difference is most likely due to the inherent antiparasitic activity of the *E. cloacae* bacterium. In addition, Wang et al. [27] reported that the expression in *P. agglomerans* of HlyA protein (which, like HasA, causes pore formation in the bacterial wall) had a negligible effect (21.2%) on parasite development. Therefore, *E. cloacae*, owing to its intrinsic antiparasitic properties could be preferred to other paratransgenesis candidates such as *Asaia* sp. and *P. agglomerans*. This advantage can be attributed to the stimulation of the mosquito's immune system and the secretion of serine protease inhibitors, which are produced by mosquitoes to control bacteria, but which are not specific to the target organism and are suppressed if the *Plasmodium* parasite is present in the midgut [49]. The *E. cloacae* bacterium is found in the normal gastrointestinal micro-flora of humans and many other animals and is generally reported to be widespread in insect midguts [43, 45, 64, 69, 70], thus alleviating any potential safety concerns concerning its release in the field.

## Conclusions

In conclusion, we consider an alternative strategy for control of the *Plasmodium* parasite that involves the use of bacterial symbionts of the mosquito, genetically engineered to express anti-*Plasmodium* effector molecules. The paratransgenesis strategy converts the proven mosquito vector into an ineffective disease vector. This approach could be effective for multiple mosquito and parasite species concomitantly. The findings of the present study provide the foundation for the use of either WT or genetically modified *E. cloacae* bacteria as a powerful tool to combat malaria. However, further studies are needed to determine how effectively these bacterial strains can be established in the field and the conditions required to do this.

## Supplementary Information

**Additional file 1: Dataset S1.** Sequences of bicistronic defensin plus green fluorescent protein (DG) construct, including the EcoRI restriction site: GATTC,—35: TTCAA (red and italic),—10: GAGACA (red and italic), ribosomal binding site number 1 (RBS1): AAAAG, signal peptide (sequences 85–135), defensin (sequences 154–309), RBS2: GAAGGAG, GFP: 305–1069, *Pst*1 restriction site: CTGCAG. Restriction sites at the beginning and end of sequences are capitalized and RBSs are underlined. Defensin and GFP are shown by cyan and green color respectively. The construct verification was performed using the following primers: forward primer DGFP1 (5’-GGA ATT CAA ATA CAT TCA AAT ATG TAT CCG-3’) and reverse primer DGFP7 (5’-TTC TGC AGT TAT TAT TTG TAT AGT TCA TCC ATG-3’).

**Additional file 2: Fig. S1.** Digestion of recombinant pBR322 containing Defensin-GFP construct (pBR/DG) and intact pBR322 plasmids with EcoRI and Pst1 restriction enzymes. M: molecular weight marker, 1: pBR/DG, 2: intact type.

## Data Availability

The datasets supporting the findings of this article are included within the article and its additional files.

## Abbreviations

CFUs: Colony-forming units
dpi: Days post-infection
GFP-D: GFP-defensin
ip: Intra-peritoneal
IRS: Indoor residual spraying
ITNs: Insecticide-treated nets
PABA: Para-aminobenzoic acid
S-HasA: Scorpine-HasA
SRPN6: Serine protease inhibitors
TBS: Transmission-blocking strategy
WT: Wild type
