# Supplementary material for: Evaluation of anti-malaria potency of wild and genetically modified Enterobacter cloacae expressing effector proteins in Anopheles stephensi
Source: Parasit Vectors. 2022 Feb 19;15:63. doi: 10.1186/s13071-022-05183-0 (PMC8858508; doi:10.1186/s13071-022-05183-0)
Supplement: Supplementary file 1 — Additional file 1: Dataset S1. Sequences of bicistronic defensin plus green fluorescent protein (DG) construct, including the EcoRI restriction site: GATTC,—35: TTCAA (red and italic),—10: GAGACA (red and italic), ribosomal binding site number 1 (RBS1): AAAAG, signal peptide (sequences 85–135), defensin (sequences 154–309), RBS2: GAAGGAG, GFP: 305–1069, Pst1 restriction site: CTGCAG. Restriction sites at the beginning and end of sequences are capitalized and RBSs are underlined. Defensin and GFP are shown by cyan and green color respectively. The construct verification was performed using the following primers: forward primer DGFP1 (5’-GGA ATT CAA ATA CAT TCA AAT ATG TAT CCG-3’) and reverse primer DGFP7 (5’-TTC TGC AGT TAT TAT TTG TAT AGT TCA TCC ATG-3’). [file 13071_2022_5183_MOESM1_ESM.docx]

**Dataset S1**

1 **GGAATTC**aaa taca***ttcaaa*** tatgtatccg ctcat***gagac a***ataaccctg ataaatgctt

61 caataatatt ga**aaaag**gaa gagtatgagt attcaacatt tccgtgtcgc ccttattccc

121 ttttttgcgg cattttgcct tcctgttttt gctcagaagt tgtgcgagag gccaagtggg

181 acatggtcag gagtctgtgg aaacaataac gcatgcaaga atcagtgcat taaccttgag

241 aaagcacgac atggatcttg caactatgtc ttcccagctc acaagtgtat ctgctacttt

301 ccttgttaac tgataaatgc ttcaataata ttaa**gaagga** **g**atatacata tgagtaaagg

361 agaagaactt ttcactggag ttgtcccaat tcttgttgaa ttagatggtg atgttaatgg

421 gcacaaattt tctgtcagtg gagagggtga aggtgatgca acatacggaa aacttaccct

481 taaatttatt tgcactactg gaaaactacc tgttccatgg ccaacacttg tcactactct

541 gacctatggt gttcaatgct tttcaagata cccagatcat atgaaacggc atgacttttt

601 caagagtgcc atgcccgaag gttatgtaca ggaaagaact atatttttca aagatgacgg

661 gaactacaag acacgtgctg aagtcaagtt tgaaggtgat acccttgtta atagaatcga

721 gttaaaaggt attgatttta aagaagatgg aaacattctt ggacacaaat tggaatacaa

781 ctataactca cacaatgtat acatcatggc agacaaacaa aagaatggaa tcaaagttaa

841 cttcaaaatt agacacaaca ttgaagatgg aagcgttcaa ctagcagacc attatcaaca

901 aaatactcca attggcgatg gccctgtcct tttaccagac aaccattacc tgtccacaca

961 atctgccctt tcgaaagatc ccaacgaaaa gagagaccac atggtccttc ttgagtttgt

1021 aacagctgct gggattacac atggcatgga tgaactatac aaataataa**C** **TGCAG**aa
