# Supplementary figures and images for: Evaluation of anti-malaria potency of wild and genetically modified Enterobacter cloacae expressing effector proteins in Anopheles stephensi
Source: Parasit Vectors. 2022 Feb 19;15:63. doi: 10.1186/s13071-022-05183-0 (PMC8858508; doi:10.1186/s13071-022-05183-0)

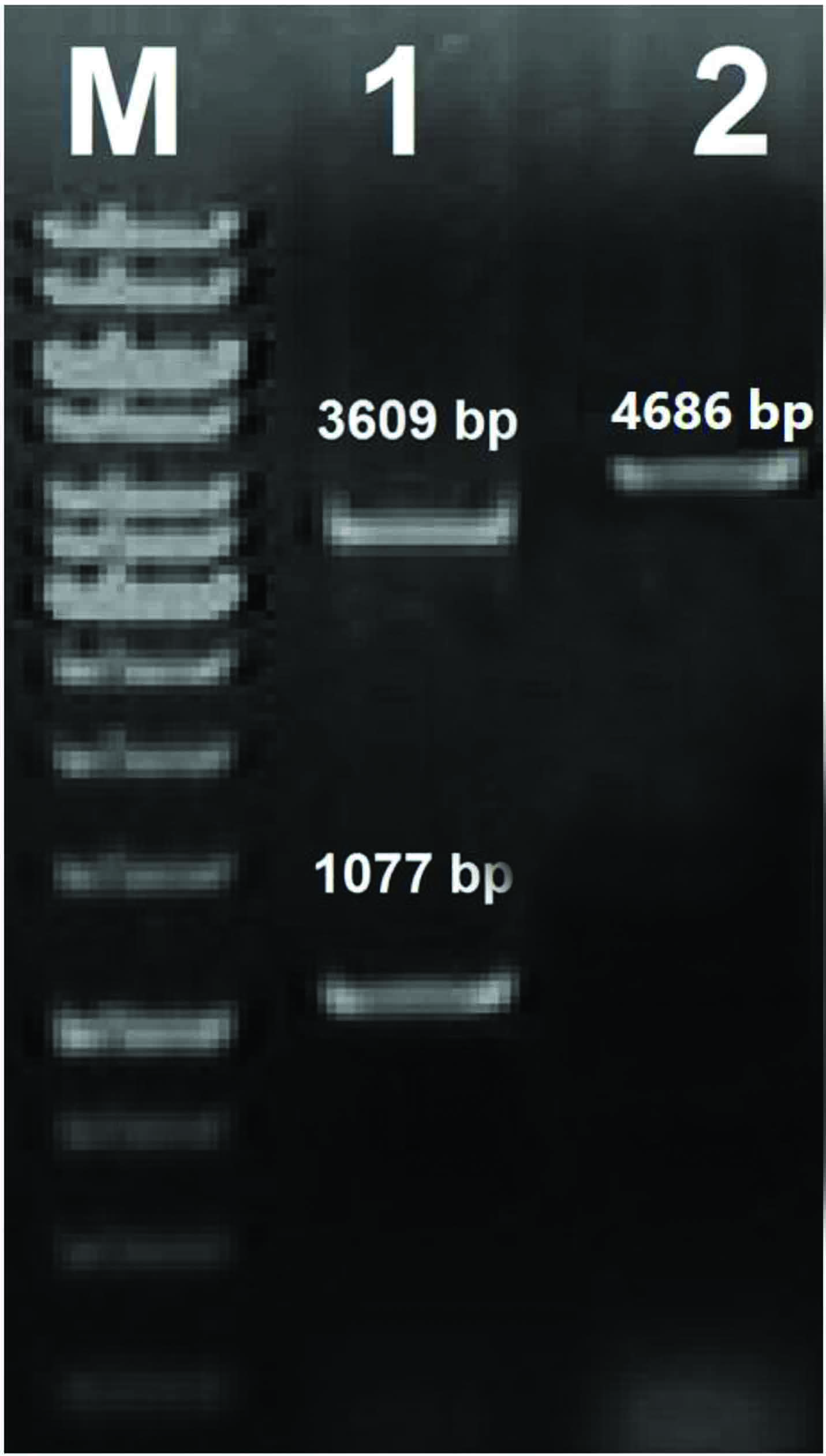

Supplement: Supplementary file 2 — Additional file 2: Fig. S1. Digestion of recombinant pBR322 containing Defensin-GFP construct (pBR/DG) and intact pBR322 plasmids with EcoRI and Pst1 restriction enzymes. M: molecular weight marker, 1: pBR/DG, 2: intact type. [file 13071_2022_5183_MOESM2_ESM.jpg]
